# Adaptively Weighted and Robust Mathematical Programming for the Discovery of Driver Gene Sets in Cancers

**DOI:** 10.1038/s41598-019-42500-7

**Published:** 2019-04-11

**Authors:** Xiaolu Xu, Pan Qin, Hong Gu, Jia Wang, Yang Wang

**Affiliations:** 1 Faculty of Electronic Information and Electrical Engineering, Dalian University of Technology, Dalian, China; 2 Department of Breast Surgery, Institute of Breast Disease, Second Hospital of Dalian Medical University, Dalian, China; 3 Institute of Cancer Stem Cell, Dalian Medical University, Dalian, China

## Abstract

High coverage and mutual exclusivity (HCME), which are considered two combinatorial properties of mutations in a collection of driver genes in cancers, have been used to develop mathematical programming models for distinguishing cancer driver gene sets. In this paper, we summarize a weak HCME pattern to justify the description of practical mutation datasets. We then present AWRMP, a method for identifying driver gene sets through the adaptive assignment of appropriate weights to gene candidates to tune the balance between coverage and mutual exclusivity. It embeds the genetic algorithm into the subsampling strategy to provide the optimization results robust against the uncertainty and noise in the data. Using biological datasets, we show that AWRMP can identify driver gene sets that satisfy the weak HCME pattern and outperform the state-of-arts methods in terms of robustness.

## Introduction

Driver mutations, which are the mutations responsible for cancer, are different from randomly occurring passenger mutations. Because driver mutations typically target genes involved in cellular signalling and regulatory pathways^1,2^. The examination of these mutations in the context of pathways and gene sets is an essential issue in cancer genome research. However, an exhaustive search for driver pathways is impossible due to the enormous number of gene set candidates. Thus, prior knowledge, such as mutation patterns, is often used as a constraint to limit the scale of the gene set candidates. In particular, high coverage and mutual exclusivity (HCME), two combinatorial properties of driver mutations in a cellular signalling pathway or regulatory pathway^2,3^, are being used as important prior knowledge in *de novo* discovery methods for driver gene sets (i.e., groups of mutated driver genes)^4–19^. High coverage means that the members in the driver gene set recurrently occur in patient cohorts, and mutual exclusivity means that almost all the patients exhibit no more than one single driver mutation event in the driver gene set. For the developments of state-of-art discovery methods for cancer driver pathways, readers are referred to the latest survey by Zhang and Zhang^20^.

The mathematical programming models for the *de novo* discovery of driver gene sets can be deduced from the HCME pattern. Vandin *et al*. developed the Dendrix algorithm, which investigates the optimal gene set by maximizing a HCME-derived score function^4^. The scoring function in Dendrix was formulated by the cardinalities of sets of patients and genes, and thus, the function was not sufficiently explicit for the optimization design. To this end, Zhao *et al*. further developed an explicit binary linear programming model and optimization framework, called MDPfinder, for the scoring system^5^. Zhao *et al*. initially introduced the genetic algorithm (GA)^21^ for this problem^5^. Leiserson *et al*. generalized Dendrix for the batch discovery of multiple driver gene sets^6^. Zhang *et al*. developed CoMDP to identify co-occurring driver gene sets^7^. Zhang *et al*. proposed ComMDP and SpeMDP to investigate common and specific driver gene sets among multiple cancer types, respectively^8^. In addition to the mathematical programming based *de novo* discovery methods, several probabilistic and statistical approaches have also been proposed. For example, Constantinescu *et al*. proposed TiMEx, a probabilistic generative model for the identification of mutually exclusive patterns^17^. Leiserson *et al*. proposed CoMEt for the identification of genes exhibiting mutual exclusivity^18^. Kim *et al*. proposed WeSME, a computational cost saving method for the permutation test in the discovery of mutual exclusivity^19^.

The assumption of mutual exclusivity implies that a patient exhibits no more than one crucial mutation event. Thus, this assumption is strong for the discovery of the driver gene sets from the mutation data of a cohort of patients. As indicated by^16^, the application of such a strong assumption can lead to a highly unbalanced pattern, in which a single frequently mutated gene is coupled to several other infrequently mutated genes to satisfy the assumption of mutual exclusivity. By observing the mutation patterns in critical cancer driver pathways (Supplementary Fig. 1), we found that a gene that is mutated in many patients always overlaps with other genes. The coverage of an individual gene is positively correlated with its overlap with other genes in a pathway. On the basis of this fact, we proposed the following weak HCME pattern for discovering a driver gene set from a cohort of patients: (a) the members in the driver gene set recurrently occur in a patient cohort; (b) the members in the driver gene set approximately satisfy mutual exclusivity and (c) the overlaps should be adequately permitted and the members that cover many patients can have relatively more overlaps than the rarely mutated members. On the other hand, the mutation datasets used in the *de novo* discovery methods are commonly sparse, i.e., the total number of patients (samples) is smaller than that of genes (variables). Similar to other data-driven inference methods, the sparseness of datasets presents another challenge for ensuring the robustness of *de novo* discovery methods.

Here, we introduce adaptively weighted and robust mathematical programming (AWRMP) for identifying driver gene sets that satisfy the weak HCME pattern. We constructed mathematical programming models using the cardinalities of sets of patients associated with the mutated genes as adaptive weights, for tuning the balance of importance between coverage and mutual exclusivity, to construct mathematical programming models. Motivated by^5^, GA^21^ was used as the basic optimization solver to efficiently solve the optimization problem. GA was embedded in a systematic subsampling strategy to obtain robust solutions against uncertainty and noise in the mutation data. Additionally, the subsampling approach can identify a parsimonious gene set, whose dimension can be considered a low bound for the dimension of the driver gene set in the sense of robustness. We applied our method to several biological datasets, and the results showed that our method identified rational driver gene sets. We then tested the significance of mutual exclusivity on our results using CoMEt^18^ and TiMEx^17^, and proved the robustness of AWRMP through a disturbance test.

## Results

### AWRMP workflow

**Figure 1:**
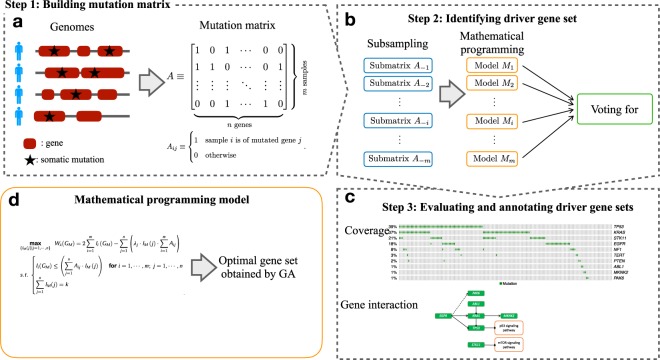
Overview of AWRMP. (**a**) We constructed binary-valued mutation matrices from mutation data files. (**b**) We used subsampling to make our method robust against the uncertainty and noise in the data. (**c**) The optimal gene set was evaluated based on coverage and exclusivity scores and annotated to analyse the gene interactions using DAVID. (**d**) We proposed a new mathematical programming model that uses adaptive weights to tune the balance between the coverage and mutual exclusivity.

The AWRMP procedure can be divided into three modules as follows (Fig. 1). We first converted the mutation data into a binary-valued matrix *A* with *m* rows (samples) and *n* columns (genes). Each element *A*_*ij*_ ∈ {0, 1} of *A* was defined as

1 $${A}_{ij}=(\begin{array}{ll}1 & {\rm{gene}}\,j\,{\rm{is}}\,{\rm{mutated}}\,{\rm{in}}\,{\rm{sample}}\,i\\ 0 & {\rm{otherwise}}\end{array}\mathrm{.}$$

We constructed a binary integer programming model on the basis of weak HCME, which is used to investigate optimal submatrix of *A*. Compared with Dendrix and its extensions, we embedded the adaptive weights to tune the balance between coverage and mutual exclusivity. GA was used as the optimization solver. We further integrated GA with a systematic subsampling strategy^22^ to eliminate the uncertainty and noise in the mutation data, and then annotated and evaluated the identified gene sets using DAVID^23^.

### Correlations between coverage score and overlap contribution

**Figure 2:**
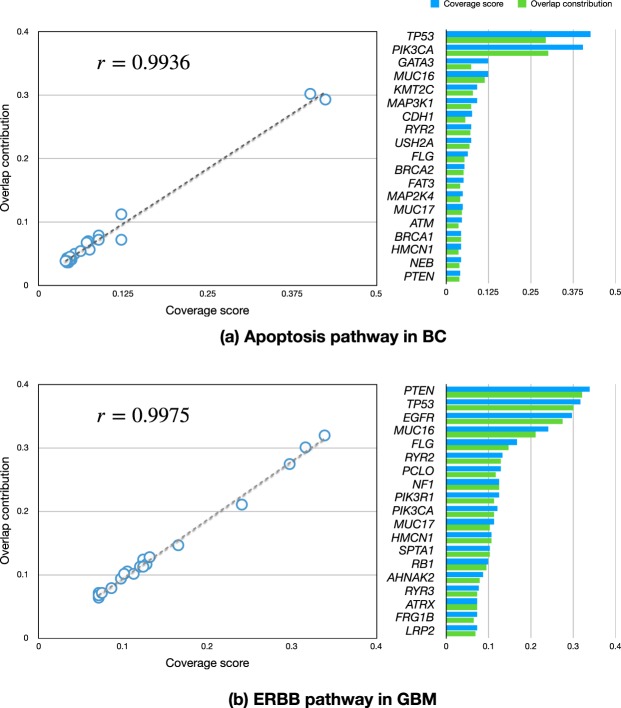
Scatter plots of the coverage score against the overlap contribution for (**a**) the apoptosis pathway of BC and (**b**) the ERBB pathway of GBM.

By observing the critical cancer driver pathways, we found that the coverage score of a mutated gene defined by formula (19) and its overlap contribution defined by formula (22) are highly positively correlated. For example, Supplementary Fig. 1 illustrates coMut plots of the somatic mutations in the apoptosis pathway obtained from the breast cancer (BC) mutation data^24^ and in the ErbB pathway obtained from glioblastoma (GBM) mutation data^25^. The two plots showed that the mutated genes approximately satisfied HCME. However, the genes with high coverage scores showed many overlaps with other genes, such as *TP53*, *PIK3CA*, *EGFR*, and *PTEN* in the two pathways. Figure 2 illustrates two scatter plots of the coverage score against the overlap contribution for all the genes in the two pathways. The correlation coefficient corresponding to the apoptosis pathway for BC was 0.9936; and that corresponding to the ErbB pathway for GBM was 0.9975. Therefore, the proper overlaps should be considered to identify the driver gene sets from mutation data from a cohort of patients.

### Identified driver gene set for lung adenocarcinoma

**Figure 3:**
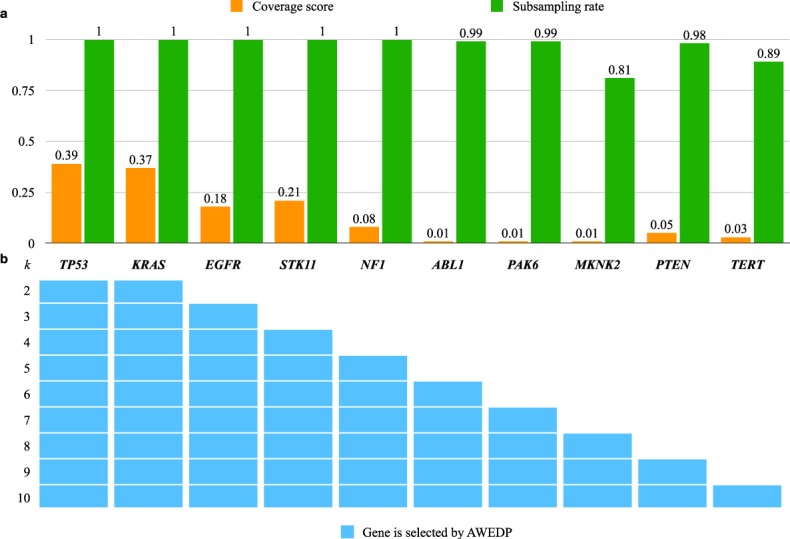
Nested gene sets identified by AWRMP for the gene set dimensions *k* = 2, 3, 4, …, 10. (**a**) Subsampling rates and coverage scores of genes obtained using Eqs (13) and (19) in Methods, respectively. (**b**) Elements of gene sets obtained with *k* = 2, 3, …, 10.

Lung adenocarcinoma (LUAD) is the most common histological type of lung cancer. To illustrate the performance of AWRMP, we applied AWRMP to LUAD mutation data^26^, which was also previoustly used to test Dendrix^4^. The variable *k* denotes the gene set dimension that is pre-defined to be identified by AWRMP. The gene sets obtained from the LUAD data with *k* = 2, 3, …, 10 were investigated (Fig. 3). *TP53*, *KRAS*, *EGFR*, and *STK11*, which have relatively high mutation frequencies, were always included in the identified gene sets obtained with *k* values larger than 4 (Fig. 3(a)). The identified gene sets became nested with increasing values of *k* (Fig. 3(b)).

For *k* = 2, the gene set (*KRAS, TP53*) was identified by AWRMP with a subsampling rate of 1 obtained using Eq. (13). In contrast, the set (*KRAS, EGFR*) was identified by Dendrix^4^. For *k* = 3, the triplet (*EGFR*, *KRAS*, *TP53*) was the unique optimal gene set selected by AWRMP with a subsampling rate 1 calculated using Eq. (13) in Methods. This gene set was mutated in 119 patients with an overlap score of 0.7059, which was obtained using Eq. (20) in Methods. Mutations in *EGFR*, *KRAS*, and *TP53* are vital in lung cancer biology, and the molecular alterations associated with these mutation profiles have been widely investigated^27^. Note that the triplet (*EGFR*, *KRAS*, *STK11*) was obtained with Dendrix. This difference was obtained because *TP53* overlapped with the other two genes, and Dendrix ignored *TP53* to ensure mutual exclusivity in its programming model.

**Figure 4:**
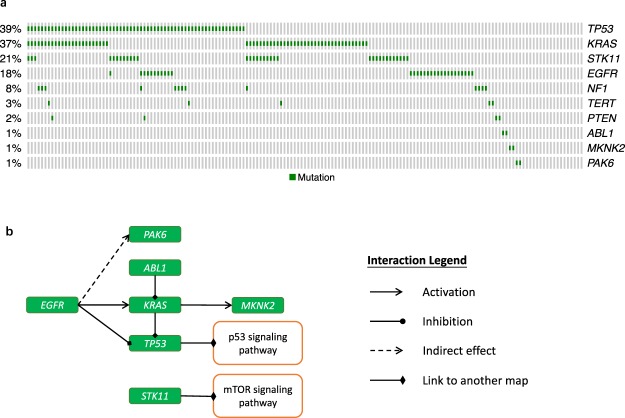
Optimal gene set identified by AWRMP for the LUAD dataset (*k* = 10). (**a**) Coverage plot of the optimal gene set. (**b**) Interaction of the genes in the optimal set annotated by knowledge of known pathways.

For *k* = 10, we found that the gene set (*ABL1*, *EGFR*, *KRAS*, *MKNK2*, *NF1*, *PAK6*, *PTEN*, *STK11*, *TERT*, *TP53*) was mutated in 145 patients (Fig. 4(a)). Through annotation using DAVID^23^, these genes were found to be involved in the ErbB, MAPK, and PI3K-Akt signalling pathways, which are known to be critical in LUAD. Based on the knowledge of these pathways, we observed that most genes in this set involve interactions (Fig. 4(b)). The subset (*KRAS*, *EGFR*, *STK11*, *PTEN*, *TP53*) covering 133 patients is a subset of the PI3K-Akt signalling pathway, and PI3K-Akt pathway mutations involved in tumourigenesis have been reported for LUAD^28^. Various treatments aiming to inhibit lung cancer cell proliferation, migration and invasion through the PI3K-Akt pathway have been developed^29^. The subset (*KRAS*, *MKNK2*, *EGFR*, *NF1*, *TP53*), which constitutes a subset of the MAPK pathway, plays a pivotal role in cell proliferation, differentiation and survival^30^. MAPK signal amplification contributes to the rapid progression of established adenomas to LUAD and takes effect during both malignant progression and tumour initiation^31,32^. The subset (*KRAS*, *MKNK2*, *EGFR*, *NF1*) overlapped in five patients, whereas the subset (*KRAS*, *MKNK2*, *EGFR*, *NF1*, *TP53*) overlapped in 44 patients. This finding indicated that *TP53* showed little mutual exclusivity with the other four genes. Whereas the remaining genes *KRAS*, *MKNK2*, *EGFR*, and *NF1* exhibited highly mutual exclusivity. The subset (*ABL1*, *EGFR*, *KRAS*, *PAK6*) which was mutated in 94 patients, forms part of the ErbB signalling pathway, which involves a family of tyrosine kinases and has been confirmed to be vital for the development of LUAD^33,34^. All the genes in this subset exhibit highly mutual exclusivity scores. Although *TERT* was not annotated in the aforementioned pathways, it has been found to be the most frequent genetic event in the early stages of non-small cell lung cancer^35^.

To date, no explicit method has been developed to determine the dimension of the driver gene set identified by *de novo* discovery method. However, based on the subsampling strategy in AWRMP, we calculated the subsampling rate of each gene using Eq. (16) in Methods. Consequently, according to the subsampling rates, the subset (*EGFR, KRAS, TP53, STK11, NF1*) can be considered a parsimonious set that shows robustness against the uncertainty and noise in the data. The dimension of the parsimonious set can be considered a lower bound for the dimension of the driver gene set.

### Performance of AWRMP

**Figure 5:**
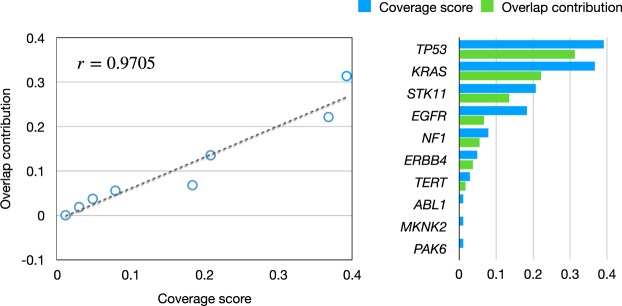
Scatter plot of coverage score against overlap contribution for the optimal gene set.

Figure 5 shows a scatter plot of the coverage score against the overlap contribution for the optimal gene set. As shown, the optimal gene set identified by AWRMP shows a similar pattern with the mutation pattern of the well-known cancer driver pathways illustrated in Fig. 2. This fact confirmed that our adaptive weights in Eq. (8) worked well, and this adaptiveness allowed us to identify useful overlaps. For example, the co-mutation (overlaps) of *TP53* and *NF1* has been known to be the feature of the PI subtype of LUAD^28^.

**Figure 6:**
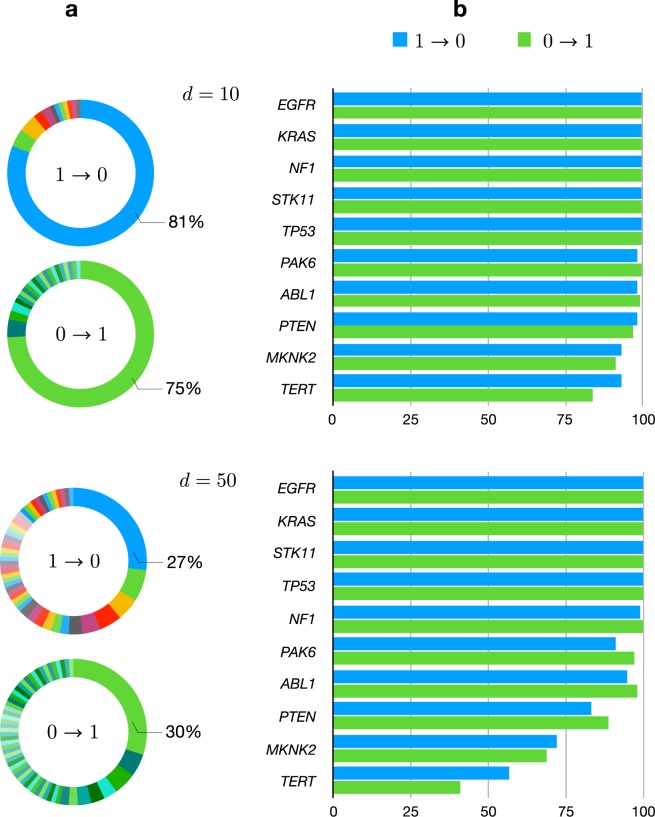
Number of times that the optimal gene set (*ABL1*, *EGFR*, *KRAS*, *MKNK2*, *NF1*, *PAK6*, *PTEN*, *STK11*, *TERT*, *TP53*) and its elements were identified by AWRMP with the 100 disturbed mutation matrices. (**a**) Percentages of the 100 disturbed mutation matrices obtained by tuning values of 1 into 0 (blue) and by tuning values of 0 into 1 (green). (**b**) Number of times that the 10 genes in the optimal gene set were identified with all 100 disturbed mutation matrices.

To illustrate the robustness of AWRMP, we artificially disturbed some elements *A*_*ij*_s of the mutation matrix *A*, by randomly turning the value 0 to 1 or randomly turning the value 1 to 0, to generate 100 new mutation matrices, and AWRMP was then performed for each disturbed mutation matrix. Consequently, the numbers of times that the candidate genes were selected by AWRMP with all 100 disturbed mutation matrices were used to evaluate the robustness of the proposed method (Fig. 6).

We conducted the disturbance test for *d* = 10 and 50, where *d* denotes the total number of disturbed elements in the mutation matrix *A*. The same optimal gene set (*ABL1*, *EGFR*, *KRAS*, *MKNK2*, *NF1*, *PAK6*, *STK11*, *TERT*, *PTEN*, *TP53*) was always identified for *d* = 10 in the both disturbance schemes (Fig. 6(a)). Increasing the value of *d* to 50, decreased the percentage of the 100 disturbed mutation matrices obtained by tuning values of 1 to 0 that yielded the optimal gene set to 27% and the percentage of the 100 disturbance schemes obtained by tuning values of 0 to 1 that yielded the optimal gene set to 30%. As expected, the robustness of AWRMP degenerated with increases in *d*. By observing the number of the times that ten genes of the optimal gene set were identified in the 100 runs of the disturbance test, we found that the subset (*EGFR*, *KRAS*, *STK11*, *TP53*, *NF1*) was always identified, even for *d* = 50 (Fig. 6(b)). This subset was thus the parsimonious set identified according to the subsampling rates. The total numbers of samples harboring these five genes were 30, 60, 34, 64, and 13, respectively. Thus the genes with relatively high coverage endured the disturbance. Furthermore, *TERT* showed the most sensitivity to the disturbance, even though it did not show the lowest observed mutation frequency. Moreover, *TERT* was not involved in any pathway detected by AWRMP (Fig. 4). This finding implies that *TERT* is slightly different from the other nine genes due to its weak HCME pattern. The results of the disturbance tests for other related methods are shown in Supplementary Fig. 2.

In addition to the robustness analysis, we also performed statistical significance tests using CoMEt^18^ and TiMEx^17^, and the results are depicted in Table 1. The optimal gene set identified by AWRMP can be considered to be significant for mutual exclusivity.

**Table 1:** Pathway enrichment analysis and assessment of the statistical significance of the optimal gene set for LUAD identified by AWRMP from LUAD mutation data.

Genes	Pathway (q-value)	CoMEt	TiMEx
*KRAS, EGFR, TP53MKNK2, NF1*	MAPK signalling pathway(2.00e-3)	0.02	6.45e-7
*KRAS, EGFR, ABL1PAK6*	ErbB signalling pathway(2.10e-3)	4.27e-8	1.57e-7
*KRAS, EGFR,STK11PTEN, TP53*	PI3K-Akt signalling pathway(4.70e-3)	0.05	3.34e-6

### Parsimonious sets identified from breast cancer and glioblastoma datasets

In addition to the LUAD data, we also applied AWRMP to mutation datasets, including BC mutation data^24^ and GBM mutation data^25^.

For the BC mutation data, AWRMP identified the parsimonious set (*AKT1, BRCA2, GATA3, MAP3K1, PIK3CA TP53, RGS1(A)*, where “(A)” refers to amplification) with a high coverage score of 0.86 and a low overlap score of *0.45* (Supplementary Fig. 3). Among these genes, *BRCA2* truncating mutations have been associated with an increased risk of BC^36^. *GATA3* has been identified as a prognostic marker for BC^37^. The genes (*AKT1, MAP3K1, PIK3CA*) are associated with the abrogation of JUN kinase signalling, which occurs in approximately half of BC patients^38^. The biological consequences of a reduction in JUN kinase activity in response to stress might include destabilization and consequent inactivation of *TP53* and thereby disruption of pro-apoptotic cellular signalling^39^. Thus, the co-mutations in the parsimonious set obtained by the adaptiveness of AWRMP are reasonable. The relation between *RGS1* mutation and BC has been discovered in^40^.

From the GBM mutation data, AWRMP identified the parsimonious set (*EGFR, NF1, PIK3CA, PIK3R1, PTEN, GABRA6, TP53*) with a coverage score of 0.70 and an overlap score of 0.30 (Supplementary Fig. 4). Among these genes, *NF1* is a human glioblastoma suppressor gene^41^, and patients harbouring *NF1* mutation or deletion tended to show decreased PKC pathway activity and elevated MAP kinase activity^25^. *GABRA6*, an inhibitory neurotransmitter in the mammalian brain, contributes to coding for a transmembrane polymorphic antigen glycoprotein^25^. The subset (*EGFR, PIK3CA, PIK3R1, PTEN, TP53*) is part of the PI3K signalling pathway, and 62% of the glioblastoma samples harboured at least one genetic event associated with this subset. The PI3K-Akt signalling pathway plays an important role in the regulation of signal transduction, which mediates various biological processes, including cell proliferation, apoptosis, metabolism, motility and angiogenesis in GBM^42^.

## Discussion

By observing the mutation patterns in cancer driver pathways from practical mutation datasets, we found the following: (a) the HCME pattern was approximately satisfied by the genes in the driver pathways and (b) overlaps were always observed, particularly among the genes with high coverage scores. For this reason, we proposed that the HCME pattern should be weakened by allowing proper overlaps in the discovery of driver gene sets. We developed AWRMP to identify the driver gene sets in cancer from mutation data. Ultimately, the goal of this approach is to investigate the gene sets that adaptively satisfy the weak HCME pattern. Moreover, by considering the sparsity of the mutation data, AWRMP can endure the potential uncertainty and noise in the data using the subsampling method. Here, we tested the performance of AWRMP using several biological datasets.

Driver mutations have often been investigated by observing the recurrence of individual genes^43,44^. However, mutational heterogeneity complicates the identification of functional mutations due to the recurrence of individual genes across many samples. As an alternative, an investigation of the putative driver gene set found across patients, has been proven to be another feasible approach. It is obvious that increases in the dimension of gene sets increases the monotonic coverage. For this reason, it becomes necessary to utilise constraints derived from biological knowledge. Notably, the mutual exclusivity of the pathways was used in combination with coverage to investigate driver gene sets. As noted by^6^, the driver pathways exhibiting the HCME patterns are generally smaller and more focused than most pathways annotated in the databases.

Figure 2 shows two examples of mutation patterns in cancer driver pathways, and these show that the coverage scores of the gene members are positively correlated with the overlap contributions. The information provided in Supplementary Fig. 6 suggests that this positive correlation can be generally observed in all mutated gene sets, not just in cancer driver pathways. Thus, when investigating cancer driver gene sets, the genes covering many patients should be allowed to exhibit more overlaps with other genes. For this reason, we claimed that the weak HCME pattern is more feasible for describing the mutation patterns in cancer driver pathways. According to the weak HCME pattern, we proposed the use of adaptive weights in AWRMP. Because of the adaptive weights included in our programming model, our results were different from those obtained with Dendrix^4^ (Supplementary Table 1), MDPfinder^5^ (Supplementary Table 2), Mutex^13^ (Supplementary Table 3), and CoMDP^7^ (Supplementary Table 4), all of which assign identical weights to all gene candidates. The analysis of LUAD mutation data using our method included *TP53* with a high coverage score in the final result. Because CoMEt and TiMEx were proposed based on the rigorously mutual exclusivity, these four related methods showed better scores than AWRMP (Supplementary Tables 7–10). However, the optimal gene set obtained by AWRMP still passed the permutation test of mutual exclusivity performed using CoMEt and TiMEx. In other words, the gene set identified by our method satisfied the mutual exclusivity, although our method permits more overlaps than other related methods. Furthermore, the overlaps identified by our AWRMP can be useful, like the overlaps between *TP53* and *NF1* identified for the LUAD data set. We do not claim that our method is better than other related approaches for the identification of *TP53*. After all, frequently mutated genes individuals can be identified using MutsigCV^43^. Our proposal is that the results obtained by AWRMP are more concordant to the objective mutation pattern, i.e., weak HCME, as demonstrated in Figs 2 and 5. Supplementary Fig. 5 shows the correlation between the coverage score and the overlap contribution of the optimal gene sets obtained by the other four methods, and these findings showed that these four methods did not satisfy the weak HCME pattern as well as our method. Note that ComMDP can also identify genes with high mutation frequencies, such as *TP53* and *PIK3CA*^8^. However, ComMDP was proposed for the identification of the common driver gene set across several types of cancer by combining their mutation matrices. Based on the mathematical programming model^8^, ComMDP is identical to MDPfinder for a single type of cancer.

In AWRMP, the optimization solver GA was embedded into the subsampling strategy to ensure the robustness of the algorithm. Prior to this study, the robustness of *de novo* discovery methods has seldom been considered. Nevertheless, the mutation matrices used as the inputs in these methods were always derived from high-throughput sequencing data, which are well known to be noisy. Furthermore, the total number of samples is notably much smaller than the number of genes. The use of sparse data always leads to statistical inference that is not robust to noise and uncertainty. The disturbance tests of Dendrix, MDPfinder, CoMDP, and, Mutex (Supplementary Tables 5 and 6, and Supplementary Fig. 2) revealed that a single run of the MCMC method and integer linear programming method were not robust to the disturbance. Because the subsampling strategy is always applied to estimate the precision of sample statistics, we adopted the subsampling method to compute the probabilities of gene sets obtained by the optimization solver. Consequently, the gene sets with high probabilities can be considered robust results. Because the adaptive weight defined by Eq. (8) is a nonlinear function of *I*_*M*_(*j*) defined by the Eq. (3), the programming model (6) is no longer a linear programming model. Motivated by^5^, the heuristic GA was used in AWRMP. As a type of combinatorial optimization model, the mathematical programming model defined by formula (8) often consists of multiple solutions. AWRMP can offer the robustness level for each solution based on the subsampling strategy.

Through AWRMP, we propose that the gene candidates should be assigned different levels of importance based on the weak HCME pattern. In addition to the weights derived from the coverage scores obtained by AWRMP, the covariates associated with mutations, such as the expression level of genes and the DNA replication time of genes used in MutsigCV^43^, can also be considered weights. The application of subsampling can assuredly increase the computational cost. However, we insist that the robust results obtained from sparse data need to be cautiously investigated.

## Methods

### Cancer genetic data and mutation matrix

We directly used the mutation matrix derived from LUAD mutation data by Dendrix^4^, which included 163 patients with at least one mutated gene and 356 genes mutated in at least one patient.

The BC and GBM mutation datasets (maf files) were downloaded from The Cancer Genome Atlas Data Portal (http://tcga-data.nci.nih.gov), and these datasets consider point mutations and copy number alterations (CNAs). Somatic point mutations were identified with MutsigCV^43^. The corresponding entry in the mutation matrix was assigned a value of 1 to indicate significant point mutation. Using the approach described in^16^, if a CNA event is concordant with the expression data, the corresponding entry in the mutation matrix is 1. After pre-processing, 487 samples and 274 genes were included in the BC mutation matrix and 282 samples and 308 genes were included in the GBM mutation matrix.

### Previous methods

For the mutation matrix *A* defined by Eq. (1), which has *m* rows (samples) and *n* columns (gene candidates), Dendrix initially proposed the following programming model for the identification of an *m* × *k* optimal submatrix *M* that satisfies the HCME pattern

2 $$W({G}_{M})\equiv |{\rm{\Gamma }}({G}_{M})|-\omega ({G}_{M})=2|{\rm{\Gamma }}({G}_{M})|-\sum _{g\in {G}_{M}}|{\rm{\Gamma }}(g)|,$$

where *G*_*M*_ denotes the set of genes corresponding to the mutation matrix *M*, Γ(*g*) ≡ {*i*: *A*_*ig*_ = 1} denotes the set of patients who presented mutations in gene *g*. *g*, *g*′ ∈ *G*_*M*_ are mutually exclusive, if Γ(*g*) ∩ Γ(*g*′) = ∅. The sum of the cardinalities ${\sum }_{g\in {G}_{M}}|{\rm{\Gamma }}(g)|$ denotes the total number of mutation events in *M*. ${\rm{\Gamma }}({G}_{M})\equiv {\cup }_{g\in {G}_{M}}{\rm{\Gamma }}(g)$ is the set of patients with mutations in the genes in *M*, and its cardinality |Γ(*G*_*M*_)| can be further used to measure the coverage of the submatrix *M*. Thus, the coverage overlap $\omega ({G}_{M})\equiv {\sum }_{g\in {G}_{M}}|{\rm{\Gamma }}(g)|-|{\rm{\Gamma }}({G}_{M})|$ can be used to measure exclusivity.

By noticing that the formula (2) is not easy for developing the optimization strategy, Zhang *et al*.^5^ initially defined two indicator functions

3 $${I}_{M}(j)\equiv (\begin{array}{ll}1 & j\in {G}_{M}\\ 0 & \,{\rm{otherwise}}\,\end{array}$$

for *j* = 1, 2, …, *n* and

4 $${I}_{i}({G}_{M})\equiv (\begin{array}{ll}1 & {\rm{genes}}\,{\rm{in}}\,{G}_{M}\,{\rm{are}}\,{\rm{mutated}}\,{\rm{in}}\,{\rm{patient}}\,i\,\\ 0 & \,{\rm{otherwise}}\,\end{array}$$

for *i* = 1, 2, …, *m*, and reformulated the maximization of *W*(*G*_*M*_) as MDPfinder, which is a binary linear programming (BLP) problem:

5 $$\begin{array}{c}\mathop{{\rm{m}}{\rm{a}}{\rm{x}}}\limits_{\{{I}_{M}(j)|j=\mathrm{1,}\cdots ,n\}}\,\,W({G}_{M})=2\sum _{i=1}^{m}{I}_{i}({G}_{M})-\sum _{j=1}^{n}({I}_{M}(j)\cdot \sum _{i=1}^{m}{A}_{ij})\\ s\mathrm{.}t\mathrm{.}\{\begin{array}{rcl}{I}_{i}({G}_{M}) & \le  & (\sum _{j=1}^{n}\,{A}_{ij}\cdot {I}_{M}(j)),\,\,{\rm{for}}\,i=\mathrm{1,}\,\cdots ,\,m;\,j=\mathrm{1,}\,\cdots ,\,n\,\\ \sum _{j=1}^{n}\,{I}_{M}(j) & = & k\end{array}\end{array}$$

### Mathematical programming model of AWRMP

In the mathematical programming model (5), *W*(*G*_*M*_) is divided into two parts: ${\sum }_{i=1}^{m}\,{I}_{i}({G}_{M})$ measures the coverage using the sum with respect to patient *i* and the second term ${\sum }_{j=1}^{n}({I}_{M}(j)\cdot {\sum }_{i=1}^{m}\,{A}_{ij})$ is the total number of mutation events (i.e., entries with a value of “1”) in the mutation matrix. The latter term indicates that MDPfinder assigns identical weights to all the genes. As we mentioned before, coverage is more important than exclusivity for the genes involved in multiple pathways. Consequently, we improve the mathematical programming model by assigning different weights to the genes contained in *G*_*M*_, i.e.,

6 $$\begin{array}{rcl}{W}_{\lambda }({G}_{M}) & \equiv  & |{\rm{\Gamma }}({G}_{M})|-{\omega }_{\lambda }(M)\\  & = & \sum _{i\mathrm{=1}}^{m}{I}_{i}({G}_{M})-(\sum _{j\mathrm{=1}}^{n}({\lambda }_{j}\cdot {I}_{M}(j)\cdot \sum _{i\mathrm{=1}}^{m}\,{A}_{ij})-\sum _{i\mathrm{=1}}^{m}\,{I}_{i}({G}_{M}))\\  & = & 2\sum _{i\mathrm{=1}}^{m}{I}_{i}({G}_{M})-(\sum _{j=1}^{n}({\lambda }_{j}\cdot {I}_{M}(j)\cdot \sum _{i\mathrm{=1}}^{m}\,{A}_{ij}))\end{array}$$

where

7 $${\lambda }_{j}\equiv (\begin{array}{ll}\frac{\exp (-|{\rm{\Gamma }}(j)|)}{\sum _{r\in {G}_{M}}\,\exp (-|{\rm{\Gamma }}(r)|)} & j\in {G}_{M}\\ 0 & \,{\rm{otherwise}}\,\end{array}$$

is the weight assigned to gene *j* for *j* = 1, 2, …, *n*. For all *j* ∈ *G*_*M*_, *λ* _*j*_∈ (0, 1) and ${\sum }_{j\in {G}_{M}}\,{\lambda }_{j}=1$. For gene *j*, *λ* _*j*_∈ (0, 1) makes coverage slightly more important than mutual exclusivity, and introduces overlaps with other genes. ${\sum }_{j\in {G}_{M}}\,{\lambda }_{j}=1$ allows the frequently mutated genes to have more overlaps than the rarely mutated genes in a gene set. In the case of *λ*_*j*_ → 1 for |Γ(*j*)|, mutual exclusivity is tuned to be as important as coverage. Using this approach, the balance between coverage and exclusivity can be adaptively adjusted for various genes with respect to the cardinality |Γ(*j*)|. For this reason, *λ*_*j*_ is called as adaptive weight. Consequently, the AWRMP programming model can be summarized as the following

8 $$\begin{array}{c}\mathop{{\rm{\max }}}\limits_{\{{I}_{M}(j)|j\mathrm{=1,}\cdots ,n\}}\,\,{W}_{\lambda }({G}_{M}\mathrm{)=2}\sum _{i\mathrm{=1}}^{m}\,{I}_{i}({G}_{M})-\sum _{j\mathrm{=1}}^{n}({\lambda }_{j}\cdot {I}_{M}(j)\cdot \sum _{i\mathrm{=1}}^{m}\,{A}_{ij})\\ s\mathrm{.}t\mathrm{.}\,\{\begin{array}{rcl}{I}_{i}({G}_{M}) & \le  & (\sum _{j=1}^{n}\,{A}_{ij}\cdot {I}_{M}(j))\,\,\,{\rm{for}}\,i=\mathrm{1,}\,\cdots ,\,m;\,j=\mathrm{1,}\,\cdots ,\,n\,\\ \sum _{j\mathrm{=1}}^{n}\,{I}_{M}(j) & = & k\end{array}\end{array}$$

### Setting up of GA

According to Eq. (7), *λ*_*j*_ is a nonlinear function of *I*_*M*_(*j*), which indicates that the AWRMP optimization model (8) is a nonlinear programming (NLP) model. According to the MDPfinder solver^5^, we used the metaheuristic GA method as the NLP solver. The settings of the GA are as follows:

#### GA search space

The genes in *A* were labeled as 1, 2, …, *n*. According to Eqs (3) and (8), a binary-valued vector ***x*** ≡ [*x*_1_, *x*_2_, …, *x*_*n*_]^Τ^ is used as an individual of a population, in which *x*_*i*_ ∈ {0, 1} characterizes the *i*-th gene in submatrix *M*. Thus, the GA search space is as follows:

9 $$S=\{{x}|{x}_{i}\in \mathrm{\{0},\,\mathrm{1\}}\,{\rm{for}}\,i=1,\,\mathrm{2,}\,\cdots ,\,n,\,\sum _{i\mathrm{=1}}^{n}\,{x}_{i}=|{G}_{M}|\}.$$

#### GA fitness function

In a GA, the fitness function is used to evaluate the quality of individual *s*_*j*_ ∈ *S*. In AWRMP, we ranked each individual solution *s*_*j*_ with respect to ${W}_{\lambda }({G}_{{M}_{j}})$ obtained by the programming model (8), in which *M*_*j*_ is the submatrix corresponding to *s*_*j*_. The ranked result, denoted by *r*_*j*_, is used to evaluate the fitness of *s*_*j*_.

#### GA operations

Selection, crossover, and mutation are three basic operators of GA. To distinguish from the above-mentioned mutation, we denoted the ‘mutation’ operator as ‘GA_mutation’. For individual *s*_*j*_ and rank *r*_*j*_ of each individual *s*_*j*_ based on the fitness value, the selection probability was defined as

10 $${p}_{j}=\frac{2{r}_{j}}{n(n+\mathrm{1)}}$$

where *n* is the population size.

The detailed GA procedure is provided in the supplementary information.

### Integrating GA with subsampling

Robustness means that the algorithm can give identical results for various datasets with high probability. Through the use of subsampling, AWRMP investigates probabilities of the gene sets selected by the GA. We used a leave-one-out subsampling strategy to obtain *n* subsamples *A*_*i*−_ for *i* = 1, 2, …, *m*, in which *A*_*i*−_ was obtained by removing the *i*th row of *A*. For all subsamples {*A*_*i*−_} and a given *k*, *m* runs of the GA were conducted to select the optimal gene sets. $\{{G}_{k}^{{\rm{SS}}}|k=\mathrm{1,}\,\mathrm{2,}\,\cdots ,\,{m}^{{\rm{SS}}}\}$ denotes the selected gene sets obtained by *m* runs of the GA. Note that the possible multiple solutions of the optimization model (8) can lead to *m*^*SS*^ > *m*. For ${G}_{k}^{{\rm{SS}}}$, we defined

11 $${m}_{k}^{{\rm{SS}}}\equiv \sum _{i\mathrm{=1}}^{m}\,{I}_{i}({G}_{k}^{{\rm{SS}}})$$

with

12 $$I({G}_{k}^{{\rm{SS}}})\equiv (\begin{array}{ll}1 & \,{G}_{k}^{{\rm{SS}}}\,{\rm{is}}\,{\rm{selected}}\,{\rm{with}}\,i{\rm{th}}\,{\rm{subsample}}\,{A}_{i-}\\ 0 & \,{\rm{otherwise}}\,\end{array}.$$

${m}_{j}^{{\rm{SS}}}$ is the total number of times that ${G}_{k}^{{\rm{SS}}}$ was selected in all *m* runs of the GA. Consequently, the probability of ${G}_{k}^{{\rm{SS}}}$ being selected as the optimal gene set can be obtained by

13 $$SS{R}_{{G}_{k}^{{\rm{SS}}}}\equiv Pr({G}_{k}^{{\rm{SS}}}\,{\rm{is}}\,{\rm{selected}})=\frac{{m}_{k}^{{\rm{SS}}}}{m}$$

which is called the subsampling rate (SSR) in this study. Moreover, the subsampling rate of a gene can also be calculated by Eq. (13), which denotes the probability of a gene being included in the optimal gene set. To test the significant robustness of ${G}_{k}^{{\rm{SS}}}$, the null hypothesis was set up as follows: the distribution of ${m}_{j}^{{\rm{SS}}}$ was assumed to be a binomial distribution Bin(*p*, *m*). By taking the uncertainty of data into consideration, *p* is further assumed to obey a Beta distribution Beta(*p*_0_*m*, *m*) where *p*_0_ ∈ (0, 1) is a user-defined hyper-parameter. In this study, *p*_0_ = 0.1. Note that the Beta distribution is a conjugate distribution of the binomial distribution and the Beta-binomial distribution is the corresponding posterior distribution. Consequently, the following statistics

14 $${Q}_{k}\equiv 1-\sum _{r=0}^{{m}_{k}^{{\rm{SS}}}}\,H(r,m,{p}_{0}m,m)$$

is calculated. *H* is the Beta-binomial probability mass function

15 $$H({m}_{1},{M}_{1},{m}_{2},{M}_{2})=(\begin{array}{c}{M}_{1}\\ {m}_{1}\end{array})\frac{B({m}_{1}+a,{M}_{1}-{m}_{1}+b)}{B(a,b)}$$

where *B*(⋅) is the Beta function, *a* = *m*_2_ + 1, and *b* = *M*_2_−*m*_2_ + 1. The ${G}_{k}^{\,{\rm{SS}}\,}$ that satisfies *Q*_*j*_ ≤ 0.05 was considered to form the driver gene set. We further defined the subsampling rate for gene *g* as follows:

16 $$SS{R}_{g}\equiv {\rm{\Pr }}(\,g\,{\rm{is}}\,{\rm{selected}}\,{\rm{in}}\,{\rm{the}}\,{\rm{driver}}\,{\rm{gene}}\,{\rm{set}})=\frac{\sum _{i\mathrm{=1}}^{m}\,{I}_{i}(g)}{m}$$

with

17 $${I}_{i}(\,g)\equiv (\begin{array}{ll}1 & \,g\,{\rm{is}}\,{\rm{selected}}\,{\rm{in}}\,{\rm{the}}\,{\rm{ith}}\,{\rm{subsampling}}\,{\rm{run}}\\ 0 & \,{\rm{otherwise}}\,\end{array}\mathrm{.}$$

Based on *SSR*_*g*_, we define a parsimonious set as follows:

18 $${\rm{Parsimonious}}\,{\rm{set}}\equiv \{g|SS{R}_{g}=1\},$$

which indicates the most robust result obtained by AWRMP.

### Evaluation of the gene set *G*

The coverage, mutual exclusivity, and optimal performance of the gene set *G* were evaluated by the coverage score, overlap score, and total score, respectively as follows:

19 $${\rm{Coverage}}\,{\rm{score}}\equiv \frac{1}{m}|{\rm{\Gamma }}(G)|$$

20 $${\rm{Overlap}}\,{\rm{score}}\equiv \frac{1}{m}\omega (G)$$

21 $${\rm{Totals}}\,{\rm{core}}\equiv \frac{1}{m}{W}_{\lambda }(G)\mathrm{.}$$

We further define the overlap contribution for gene *g* ∈ *G* as follows:

22 $${\rm{Overlap}}\,{\rm{contribution}}\,{\rm{of}}\,{\rm{gene}}\,g\equiv \frac{1}{m}(\omega (G)-\omega ({G}_{g-}))$$

where *G*_*g*−_ is the gene set obtained by subtracting gene *g* from gene set *G*, and this analysis is used to measure how gene *g* affects the overlap score of *G*.

## Supplementary information

SUPPLEMENTARY INFORMATION FOR “ADAPTIVELY WEIGHTED AND ROBUST MATHEMATICAL PROGRAMMING FOR THE DISCOVERY OF DRIVER GENE SETS IN CANCERS”
