# Supplementary material for: Adaptively Weighted and Robust Mathematical Programming for the Discovery of Driver Gene Sets in Cancers
Source: Sci Rep. 2019 Apr 11;9:5959. doi: 10.1038/s41598-019-42500-7 (PMC6459865; doi:10.1038/s41598-019-42500-7)
Supplement: Supplementary file 1 — SUPPLEMENTARY INFORMATION FOR “ADAPTIVELY WEIGHTED AND ROBUST MATHEMATICAL PROGRAMMING FOR THE DISCOVERY OF DRIVER GENE SETS IN CANCERS” [file 41598_2019_42500_MOESM1_ESM.pdf]

SUPPLEMENTARY INFORMATION FOR "ADAPTIVELY WEIGHTED AND ROBUST MATHEMATICAL PROGRAMMING FOR THE DISCOVERY OF DRIVER GENE SETS IN CANCERS"

XIAOLU XU[1], PAN QIN[1], HONG GU[1], JIA WANG[2,*], AND YANG WANG[3]

1. Tables

Table 1. Driver gene sets for LUAD mutation data obtained by Dendrix: The subsampling yielded 163 datasets. Dendrix was run with default parameters, and the gene sets identified by Dendrix with the largest subsampling rate were taken as the driver gene sets.

k	Genes	Subsampling rate
2	*EGFR, KRAS*	1
3	*EGFR, KRAS, STK11*	1
4	*EGFR, EPHB1, KRAS, STK11*	0.81
5	*EGFR, FES, KRAS, PRKCG, STK11*	0.06
6	*ABL1, EGFR, KRAS, PRKCG, STK11, TERT*	0.01
7	*EGFR, JUP, KRAS, MAP3K3, MATK, STK11, TFDP1*	0.006
8	*EGFR, FES, HD, KRAS, MAP3K15, MEN1, PRKCG, STK11*	0.006
9	*ABL1, BRCA1, CYSLTR2, EGFR, EPHB1, KRAS, MATK, PTK2, STK11*	0.006
10	*EGFR, EVI1, KIAA1303, KRAS, MATK, MKNK2, PRKCG, RPS6KA1, STK11, STK38L*	0.006

Table 2. Driver gene sets for LUAD mutation data obtained by MDPfinder: The subsampling yielded 163 datasets. The BLP model of MDPfinder was run with default parameters, and the gene sets identified by MDPfinder with the largest subsampling rate were taken as the driver gene sets.

k	Genes	Subsampling rate
2	*EGFR, KRAS*	1
3	*EGFR, KRAS, STK11*	0.59
4	*EGFR, KRAS, PRKCG, STK11*	0.84
5	*EGFR, EPHB1, KRAS, MAP3K3, STK11*	0.93
6	*EGFR, EPHB1, KRAS, MAP3K3, PAK6, STK11*	0.53
7	*ABL1, CYSLTR2, EGFR, EPHB1, KRAS, MAP3K3, STK11*	0.24
8	*ABL1, CYSLTR2, EGFR, EPHB1, KRAS, MAP3K3, PAK6, STK11*	0.31
9	*ABL1, CYSLTR2, EGFR, EPHB1, FES, KRAS, MAP3K3, PAK6, STK11*	0.61
10	*ABL1, CYSLTR2, EGFR, EPHB1, FES, JUP, KRAS, MAP3K3, PAK6, STK11*	0.55

Table 3. Driver gene sets for LUAD mutation data obtained by Mutex: The subsampling yielded 163 datasets. Mutex was run with the maximum group size from 2 to 10 and with default permutation parameters (first level random iteration 10000, second level random iteration 0). The gene sets with the minimum p-value (less than 0.1) and maximum size were taken as the driver gene sets.

k	Genes	Subsampling rate
2	*EGFR, KRAS*	1.00
3	*EGFR, EPHB1, KRAS*	0.90
4	*EGFR, KRAS, NRAS, PRKCG*	0.70
5	*EGFR, EPHB1, KRAS, MAP3K3, STK11*	0.90
6	*EGFR, KRAS, MAP3K3, NF1, PTK2, STK11*	0.70
7	*ABL1, EGFR, KRAS, MAP3K3, NF1, PTK2 STK11*	0.40
8	*ABL1, EGFR, ERBB4, KRAS, MAP3K3, NF1, PTK2 ,STK11*	0.50
9	*ABL1, EGFR, ERBB4, KRAS, MAP3K3, NF1, PAK6, PTK2, STK11*	0.60
10	*ABL1, EGFR, ERBB4, KRAS, MAP3K3, MAST1, NF1, PAK6, PTK2, STK11*	0.40

Table 4. Driver gene sets for LUAD mutation data obtained by CoMDP: The subsampling yielded 163 datasets. CoMDP was run with $\lambda = -10$ and $\eta = 2$ to detect gene sets with small common coverage. The combined gene sets identified by CoMDP with the largest subsampling rate were taken as the driver gene sets.

k	Genes	Subsampling rate
2	*EGFR, KRAS*	1.00
3	*EGFR, KRAS, STK11*	1.00
4	*EGFR, KRAS, NF1, STK11*	1.00
5	*EGFR, ERBB4, KRAS, NF1, STK11*	0.97
6	*MAP3K3, EGFR, ERBB4, KRAS, NF1, STK11*	0.45
7	*ABL1, EGFR, ERBB4, KRAS, MAP3K3, NF1, STK11*	0.18
8	*ABL1, EGFR, ERBB4, KRAS, MAP3K3, MAST1, NF1, STK11*	0.10
9	*ABL1, EGFR, ERBB4, KRAS, MAP3K3, MKNK2, NF1, PAK6, STK11*	0.06
10	*PTK2, ABL1, EGFR, ERBB4, KRAS, MAP3K3, MAST1, MKNK2, NF1, STK11*	0.06

Table 5. Ten elements disturbance tests for Dendrix, MDPfinder, Mutex, CoMDP and AWRMP for LUAD mutation data

Method	Disturbance	Genes	Frequencies	Coverage score	Overlap score
Dendrix	0 to 1	*EGFR,EPHB1,GNAS,HCK,KRAS,PAK6 PIK3C2B,RPSA,STK11,VAV2*	1%	0.77	0.11
MDPfinder	0 to 1	*ABL1,CYSLTR2,EGFR,EPHB1,FES,JUP KRAS,MAP3K3,PAK6,STK11*	12%	0.78	0.09
Mutex	0 to 1	*ABL1, EGFR, ERBB4, KRAS, MAP3K3 MAST1, MKNK2, NF1, PAK6, STK11*	50%	0.82	0.13
CoMDP	0 to 1	*ABL1, EGFR, ERBB4, KRAS, NF1, STK11 CYSLTR2, MAP3K3, PAK6, PTK2*	2%	0.82	0.13
AWRMP	0 to 1	*ABL1,EGFR,KRAS,MKNK2,NF1,PAK6 PTEN,STK11,TERT,TP53*	75%	0.89	0.44
Dendrix	1 to 0	*ABL1,AURKA,BAX,EGFR,ERBB4,KRAS MKNK2,PRKCG,STK11,TNK2*	1%	0.77	0.10
MDPfinder	1 to 0	*ABL1,CYSLTR2,EGFR,EPHB1,FES,KRAS MAP3K3,PAK6,RPS6KA6,STK11*	13%	0.78	0.09
Mutex	1 to 0	*ABL1, EGFR, ERBB4, KRAS, MAP3K3 MAST1, NF1, PAK6, PTK2, STK11*	20%	0.82	0.13
CoMDP	1 to 0	*MAP3K3, MAST1, PTK2, ABL1, EGFR ERBB4, KRAS, NF1, PAK6, STK11*	3%	0.82	0.13
AWRMP	1 to 0	*ABL1,EGFR,KRAS,MKNK2,NF1,PAK6 PTEN,STK11,TERT,TP53*	81%	0.89	0.44

TABLE 6. Fifty elements disturbance tests for Dendrix, MDPfinder, Mutex, CoMDP and AWRMP for LUAD mutation data

Method	Disturbance	Genes	Frequencies	Coverage score	Overlap score
Dendrix	0 to 1	*EGFR,FES,JUP,KRAS,MAP3K15 MAP3K3,MYCN,STK11,STK36,TNK2*	1%	0.75	0.09
MDPfinder	0 to 1	*ABL1, EGFR, EPHB1, FES, KRAS MAP3K3,MAST1,PAK6,RPS6KA6,STK11*	3%	0.78	0.09
Mutex	0 to 1	*ABL1,EGFR,ERBB4,KRAS,MAP3K3 MAST1, MKNK2, NF1, PAK6, STK11*	40%	0.82	0.13
CoMDP	0 to 1	*ABL1, MAST1, MKNK2, PTK2, EGFR ERBB4, KRAS, MAP3K3, NF1, STK11*	2%	0.82	0.13
AWRMP	0 to 1	*ABL1,EGFR,KRAS,MKNK2,NF1,PAK6 PTEN,STK11,TERT,TP53*	30%	0.89	0.44
Dendrix	1 to 0	*BUB1,EGFR,EPHA6,FYN,KRAS,MAP3K3 PFTK1,PIK3C2B,PLCG2,STK11*	1%	0.74	0.11
MDPfinder	1 to 0	*ABL1,CYSLTR2,EGFR,EPHB1,FES,KRAS MAP3K3,MAST1,PAK6,STK11*	2%	0.78	0.09
Mutex	1 to 0	*ABL1, EGFR, ERBB4, KRAS, MAP3K3 MAST1, NF1, PAK6, PTK2, STK11*	20%	0.82	0.13
CoMDP	1 to 0	*ABL1, CYSLTR2, EGFR, EPHB1, GNAS KRAS, MAP3K3, NF1, PAK6, STK11*	2%	0.82	0.14
AWRMP	1 to 0	*ABL1,EGFR,KRAS,MKNK2,NF1,PAK6 PTEN,STK11,TERT,TP53*	27%	0.89	0.44

Table 7. The pathway enrichment analysis and statistical significance test for the optimal gene set for LUAD mutation data identified by Dendrix.

**Genes**	**Pathway (q-value)**	**CoMEt**	**TiMEx**
KRAS,MKNK2,EGFR,PRKCG,RPS6KA1	MAPK signaling pathway (2.20e-3)	2.31e-10	4.38e-19
PRKCG,RPS6KA1,STK11	mTOR signaling pathway (3.00e-2)	0.15	0.03

Table 8. The pathway enrichment analysis and statistical significance test for the optimal gene set for LUAD mutation data identified by MDPfinder.

**Genes**	**Pathway (q-value)**	**CoMEt**	**TiMEx**
ABL1,EPHB1,FES,KRAS,PAK6	Axon guidance (9.10e-04)	3.42e-3	1.05e-5
ABL1,KRAS,EGFR,PAK6	ErbB signaling pathway (5.40e-03)	3.08e-9	3.49e-17

Table 9. The pathway enrichment analysis and statistical significance test for the optimal gene set for LUAD mutation data identified by Mutex.

**Genes**	**Pathway (q-value)**	**CoMEt**	**TiMEx**
ABL1,KRAS,EGFR,ERBB4,PAK6,PTK2	ErbB signaling pathway (1.10e-6)	7.93e-9	5.46e-17
ABL1,KRAS,EGFR,NF1,PAK6	Ras signaling pathway (2.60e-3)	1.00e-7	2.25e-15
KRAS,EGFR,ERBB4,PTK2	Proteoglycans in cancer (2.20e-2)	2.14e-7	1.46e-14

Table 10. The pathway enrichment analysis and statistical significance test for the optimal gene set for LUAD mutation data identified by CoMDP.

**Genes**	**Pathway (q-value)**	**CoMEt**	**TiMEx**
ABL1,KRAS,EGFR,ERBB4,PTK2	ErbB signaling pathway (1.20e-4)	4.27e-8	9.28e-16
KRAS,MKNK2,EGFR,MAP3K3,NF1	MAPK signaling pathway (4.00e-3)	4.43e-8	5.66e-16

2. Figures

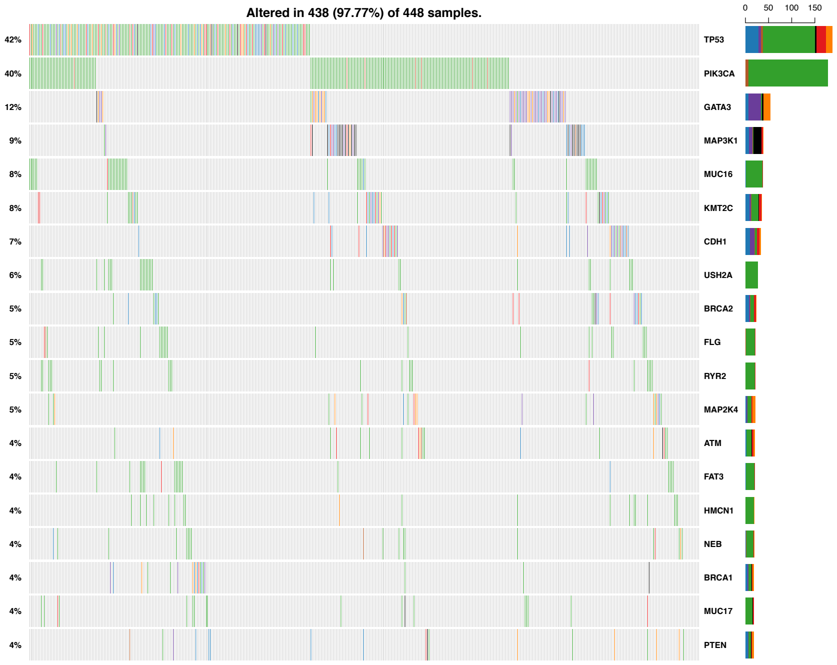

(a) Apoptosis pathway in BC

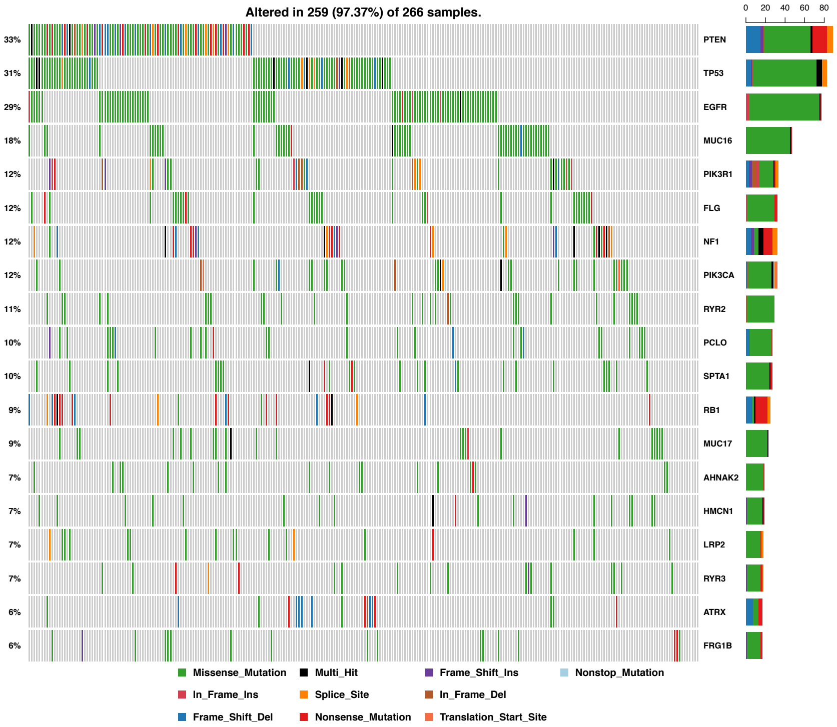

(b) ERBB pathway in GBM

Figure 1. The coMut plots of significantly mutated genes for (a) Apoptosis pathway in BC; (b) ERBB pathway in GBM. All three data sets are downloaded from TCGA. The genes in the driver pathways approximately satisfy the high coverage and mutual exclusive pattern.

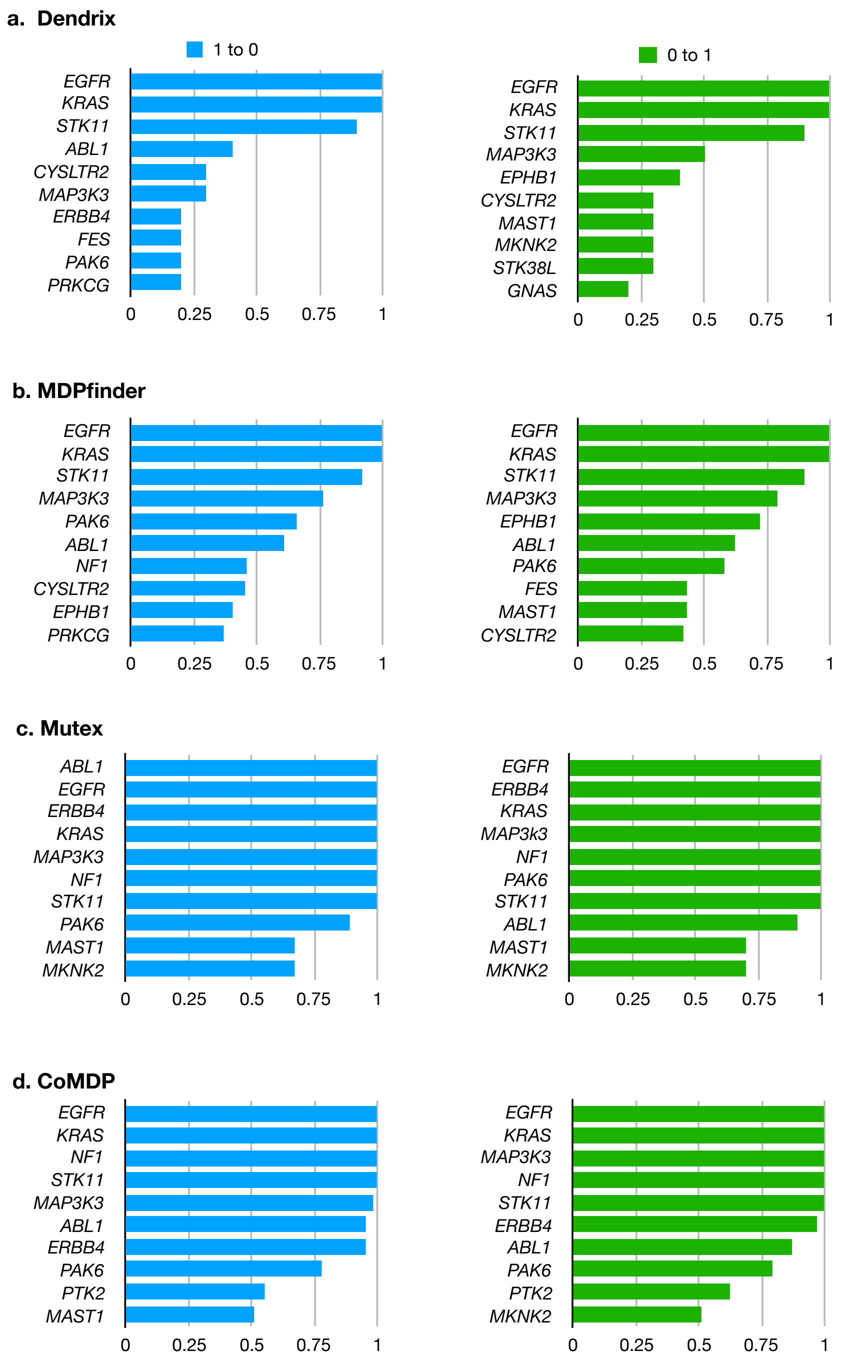

FIGURE 2. The ten most robust genes identified by the ten elements disturbance tests of Dendrix and MDPfinder. To illustrate the robustness of the identified genes, we use the frequencies of genes included in the optimal genes sets identified by Dendrix, MDPfinder, Mutex, and CoMDP for all the disturbed mutation matrices.

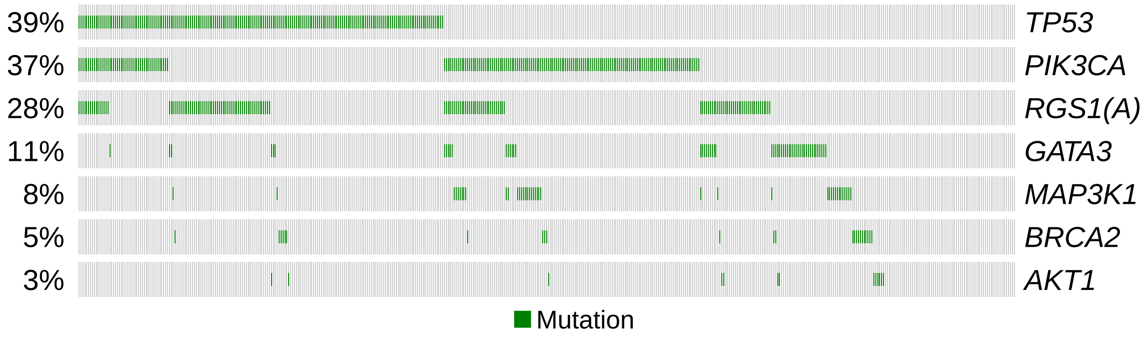

FIGURE 3. The coverage plot of the parsimonious gene set identified for the BC mutation data.

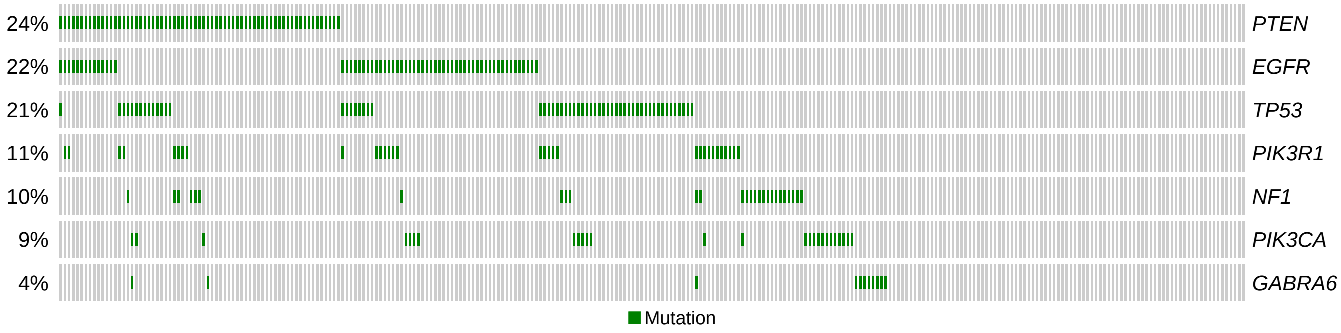

FIGURE 4. The coverage plot of the parsimonious gene set identified for the GBM mutation data.

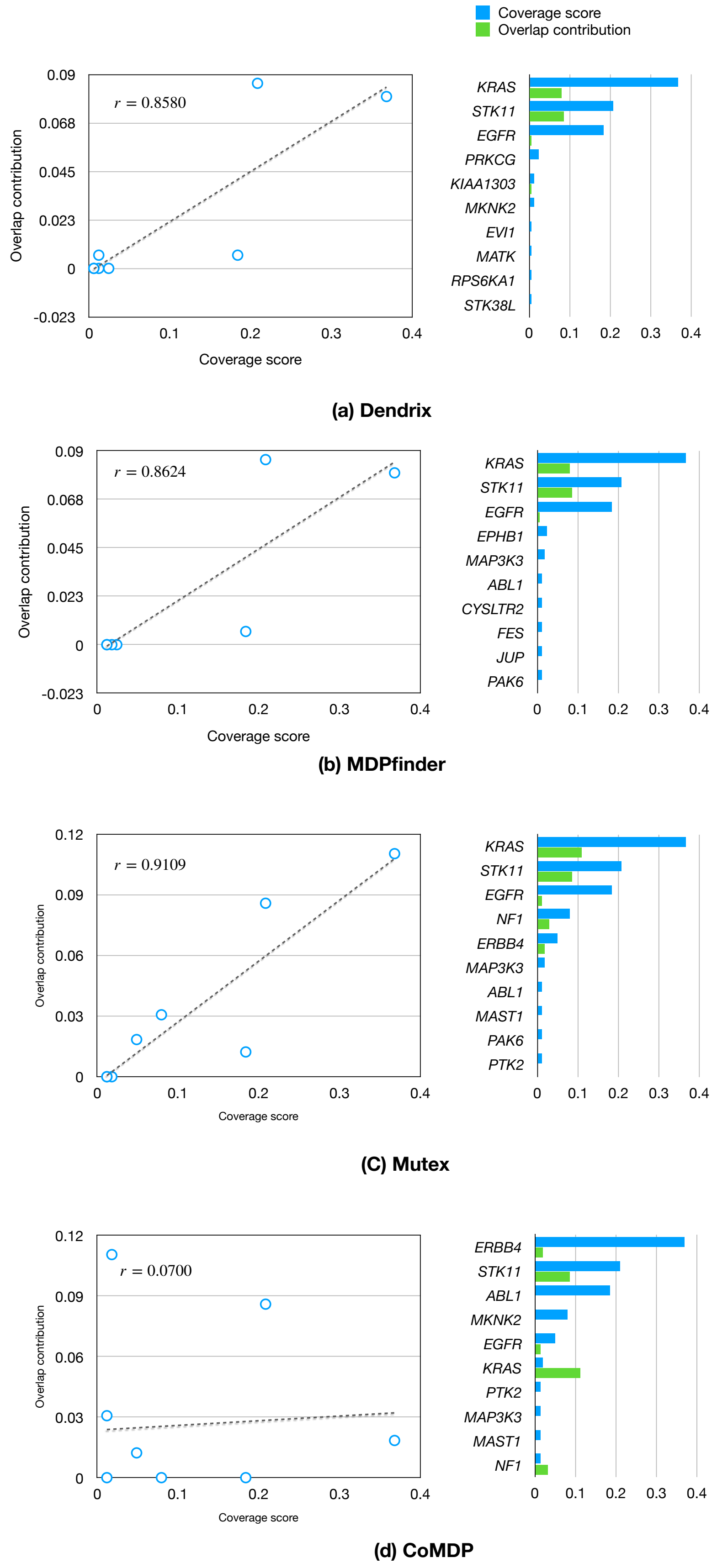

FIGURE 5. Scatter plots of coverage score against overlap distribution for the optimal gene sets obtained in Supplementary Tables 1∼4 by Dendrix, MDPfinder, Mutex, and CoMDP.

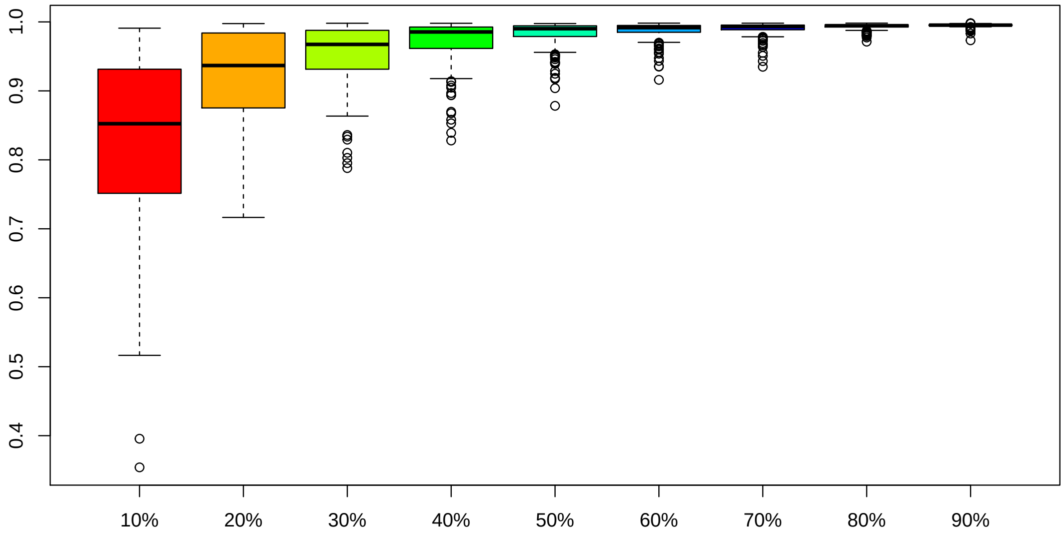

FIGURE 6. Box plot shows the five-number summary of correlation coefficients for coverage scores against overlap contributions obtained from randomly sampled gene subsets in the LUAD data set. The gene subsets were randomly drawn from the LUAD dataset according to uniform distributions for 200 independent runs. The coverage scores and overlap contributions were calculated for the genes in each subset and hereby the correlation coefficients were obtained. The sizes of subsets were prefixed as 10% to 90% of a total of 365 mutated genes in LUAD dataset. The results suggested that the coverage scores and overlap contributions of mutated genes in a gene set are often positively correlated.

3. GA procedure

GA used for AWRMP can be described as follows:

S-1: Initializations. Iteration number $t = 300$; population size $P = 200$; gene set size $2 \leq k \leq 10$; reproduction probability $p_j = \frac{2r_j}{n(n+1)}$; GA_mutation rate $p_m = 0.1$.

S-2: Selection of the optimal individual. Calculate the fitness of individuals through weight submatrix function $W_\lambda(M_j)$ and determine an order r_j by ranking each individual s_j based on fitness value. If the termination criteria is reached, output the best individual and the corresponding optimal gene set, else proceed to next step. The individuals with high fitness are selected with high probability, and the individuals with low fitness are eliminated.

S-3: Crossover. P new individuals $s'_1, s'_2, \cdots, s'_P$ are generated by P couples $s_1, s_2, \cdots, s_{2P}$ from current population.

S-4: GA_mutation. Several new individuals are generated according to a certain variation rate $p_m = 0.1$.

S-5: Avoid trapping local solution. Check whether the algorithm falls into the local optimum, that is , the maximum fitness does not change within 5 iterations. If so, reset individuals by GA_mutation, and go back to S-2.

S-6: Next generation. A new population of individuals is produced by the selection, crossover and GA_mutation. The best P individuals are chosen to form the next generation, and go back to S-2.

[1]Faculty of Electronic Information and Electrical Engineering, Dalian University of Technology

[2]Department of Breast Surgery, Institute of Breast Disease, Second Hospital of Dalian Medical University

[3] Institute of Cancer Stem Cell, Dalian Medical University
